# Evaluation of Physiotherapy Impact on Neuromuscular Tension in Analog Astronauts at the LunAres Habitat

**DOI:** 10.3390/ijerph19116888

**Published:** 2022-06-04

**Authors:** Barbara Gronwald, Karina Kijak, Piotr Baszuk, Danuta Lietz-Kijak, Kamil Kosko, Mikołaj Matuszczak, Piotr Skomro, Hanna Bielawska-Victorini, Leszek Orzechowski, Agata Mintus, Helena Gronwald

**Affiliations:** 1 Department of Propaedeutics, Physical Diagnostics and Dental Physiotherapy, Pomeranian Medical University in Szczecin, 71-252 Szczecin, Poland; 2 Research Club STO-MATER-FIZ, Department of Propaedeutics, Physical Diagnostics and Dental Physiotherapy, Pomeranian Medical University in Szczecin, 71-252 Szczecin, Poland; karo0326@gmail.com (K.K.); mikolaj.matuszczak@gmail.com (M.M.); 3 Department of Genetics and Pathology, Pomeranian Medical University in Szczecin, 71-252 Szczecin, Poland; piotr.baszuk@pum.edu.pl; 4 Department of Propaedeutics, Physical Diagnostics and Dental Physiotherapy, Pomeranian Medical University in Szczecin, 71-252 Szczecin, Poland; danuta.lietzkijak@gmail.com (D.L.-K.); skomro@froe.pl (P.S.); helenagronwald@gmail.com (H.G.); 5 Individual Dental Practice Kamil Kosko, 62-510 Konin, Poland; kamil.kosko@gmail.com; 6 Department of Orthodontics, Pomeranian Medical University in Szczecin, 71-252 Szczecin, Poland; hania.bielawska@wp.pl; 7 LunAres Research Station, 64-930 Piła, Poland; orzechleszek@gmail.com (L.O.); agata.mintus@gmail.com (A.M.)

**Keywords:** TMD, analog space mission, stress, isolation, trigger point therapy

## Abstract

The evaluation of manual Trigger Point Therapy (TrPt) on mandible abduction range of Analog Astronauts (AA) surviving isolation conditions during consecutive missions at the LunAres Habitat was performed. This physiotherapy method was applied to decrease stress-related neuromuscular tension. Abduction measurements were conducted on the two groups of five AA, who endured severe isolation conditions for 14 days in the limited space of the LunAres Research Station Habitat (Piła, Poland) during missions. The test group consisted of abduction measurements of AA who received TrPt and control group of abduction measurements of AA who did not receive TrPt. All measurements were noted in the TemporoMandibular Joint (TMJ) diagnosis aspect of the integrated dental examination card SZOPPDP©. The ischemic compression was performed on an active localized trigger point—resulting in cessation of pain. Maximum abduction measurements were made with an electronic caliper, and the abduction range was compared. The change of abduction range in AA with TrPt was bigger than in AA without TrPt. A larger increase in abduction range was observed in every case in the group receiving TrPt compared to the control group. TrPt effectively decreases the neuromuscular tension, which results in an increased mandibular abduction range of AA. Observations conducted in LunAres Research Station regarding stress-related neuromuscular tension can help identify effective therapeutic methods for circumstances of social isolation.

## 1. Introduction

Individuals who endure some forms of isolation have increased levels of stress [1,2,3]. It was shown that stress is associated with increased neuromuscular tension and can affect muscle function [4]. Investigation of possible effective methods to diminish stress could have positive effects on people affected by long-term stress.

**Figure 1:**
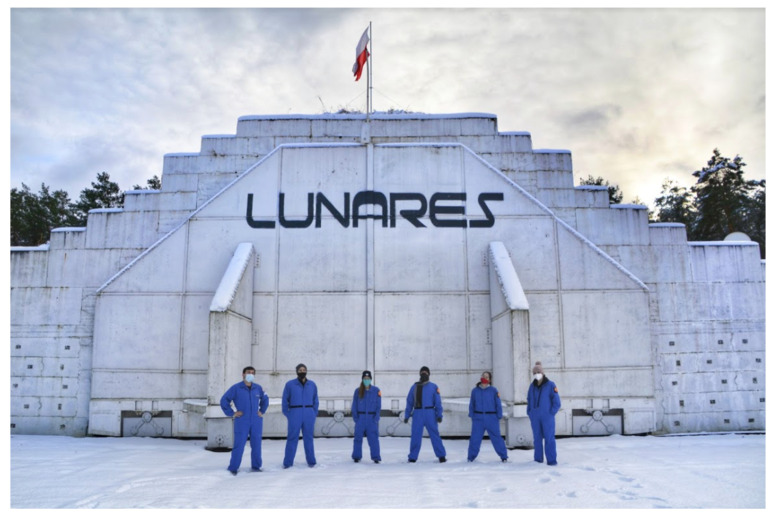
Crew of Analog Astronauts at LunAres Research Station (Piła, Poland). Authorship: LunAres Research Station.

Research with numerous variables that can affect the tested parameters is very difficult to plan and conduct. An example of such an attempt is to investigate the impact of longstanding stress on human physiology. For the sake of accidental factors elimination, such studies are often carried out in special habitats simulating a particular reality [5]. One such habitat is the LunAres Research Station, a facility used to simulate manned Martian and Lunar missions (Figure 1).

LunAres was built in 2017 and was the first of its kind in Europe. This station was created to become a platform where researchers could study different aspects of manned extra-terrestrial exploration as well as develop, test, and upgrade various technologies that could be useful in space or help to better prepare astronauts before their missions. Due to its interdisciplinary research drive, the facility provides a unique opportunity to conduct experiments that normally would be difficult to investigate, especially ensuring the safety of everyone involved. Specialists from various fields are involved in the study of extreme environments, sustainability, extreme plant cultivation, biotechnology, robotics, engineering, sociology, psychology, medicine, and even space architecture. The influence of simulated conditions of Mars and the Moon on completely isolated analog astronauts can be studied in the facility thanks to the possibility to constantly observe the inside of the base, directly and indirectly, control the internal environment, and access the telemetry of the physical and psychological states of the crew.

This also provides an opportunity to create and test proper health and safety procedures and find solutions to problems that could arise during an actual space mission without endangering any participants. This makes the analog space base a core element in pioneering space research in addition to preparation for extra-terrestrial expeditions.

**Figure 2:**
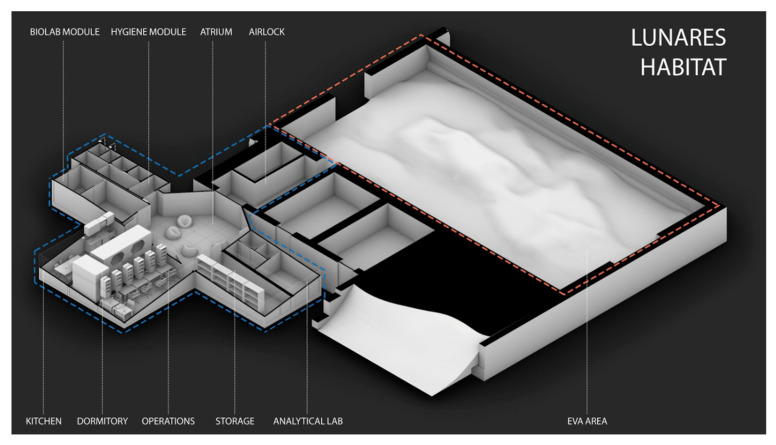
Infrastructure of LunAres Research Station Habitat in Piła, Poland. Authorship: LunAres Research Station.

LunAres Research Station is located at the post-military airport in Piła, Poland. The facility provides complete isolation of analog base/living module and a very large Extravehicular Activity area (250 m^2^) from the external environment. The capacity of the base allows for organizing 2-week missions for crews of six people maximum. The behavior and health parameters of the AA are constantly monitored thanks to the stations’ infrastructure (Figure 2).

Conducting research in such a unique facility gives the opportunity to closely inspect the impact of different factors that are not easily researchable in everyday life, such as the influence of isolation or stress on the stomatognathic system. During two missions of the study, the main interest was the isolation-related stress, neuromuscular tension, and possible temporomandibular disorder (TMD) diagnosis due to the possible isolation-induced stress and impacted neuromuscular tension.

Manual Trigger Point Therapy (TrPt) is one of the therapeutic methods of relieving trigger points (TrP) created within muscles and fascia mostly in response to increased neuromuscular tension due to stress. The reduction of the tension causes the muscle to relax and increase its working range [6]. TrPt was selected due to its recognized technique [7,8] as well as the possibility of conducting it in isolated conditions.

In order to record and better diagnose all aspects of a patient’s masticatory system, an integrated dental examination card SZOPPDP© was used. Integrated dental examination card SZOPPDP© (Polish abbreviation translated: S, Temporomandibular Joint Dysfunction/Disorder; Z, Conservative Dentistry; O, Orthodontics; P, Periodontology; P, Oral Prophylaxis; D, Diet; P, Psychology) is a diagnostic and survey tool for defining the risk level for individual diseases and habits that could impact oral health. This holistic diagnostic approach enables prescribing to the patient precise personal recommendations based on the latest guidelines from the fields of dentistry and medicine. Does the manual TrPt influence the scope of mandibular abduction in the conditions of analog space station isolation, and, if so, what is the impact? Can a change in the extent of mandibular abduction range be a marker that facilitates the diagnosis of patients with increased risk of TMD? This study aimed to assess the impact of the manual TrPt on the mandible abduction range in order to decrease stress-related neuromuscular tension and diagnose patients with increased TMD risk using the integrated dental examination card SZOPPDP© in AA surviving isolation conditions during consecutive missions at the LunAres Habitat.

## 2. Materials and Methods

The study was conducted at LunAres Research Station (Piła, Poland). Harsh isolation conditions were integrated and researched during every analog astronaut mission in LunAres, including:

- No access to the sunlight (time of the day was simulated with artificial lighting only);
- Strict prohibition to leaving the facility during the 14-day mission;
- Contact with an external environment limited to an absolute emergency;
- Experiencing decreased physical activity.

The study was conducted on the 2 groups of 5 AA (Table 1), who endured severe isolation conditions for 14 days in the limited space of the LunAres Habitat during missions as a part of the Pandemic Isolation Campaign in 2021:

- Test group: maximal mandibular abduction measurements of AA who received TrPt during their mission.
- Control group: maximal mandibular abduction measurements of AA who did not receive TrPt.

**Figure 3:**
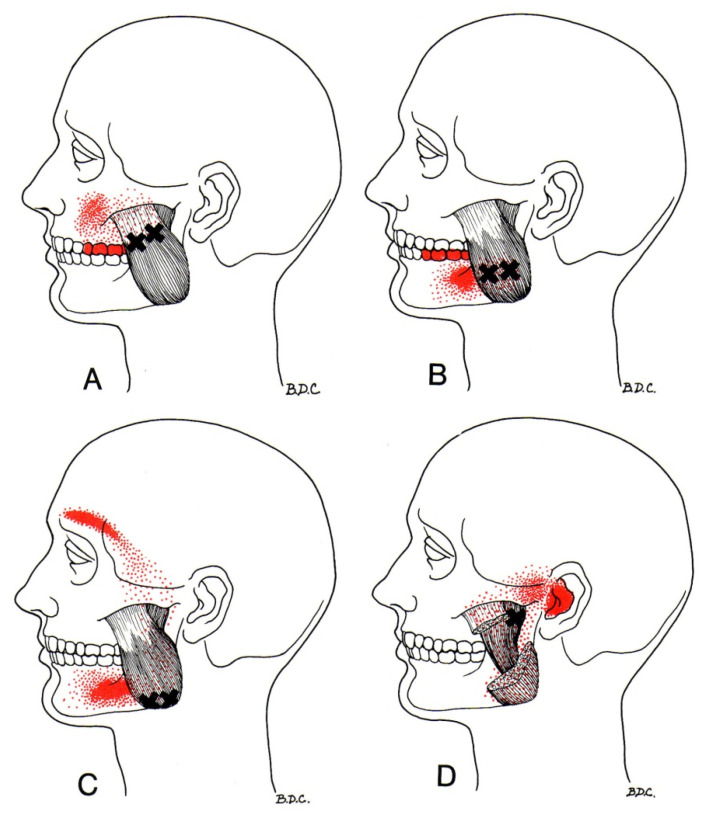
(**A**–**D**) Localization of Trigger Points (black crosses) of Masseter Muscle and areas of referred pain (red areas). Authorship: www.triggerpoints.net (Access date: 16 March 2021).

Trigger Point Therapy was performed in the area of upper and lower attachments and the belly of the masseter muscle on the right and left side with a closed mandible [7] (Figure 3).

To diagnose the active TrP, a pincer grip was used between the thumb and pointer to embrace the tensed tissue from both sides. The ischemic compression was performed on the active localized trigger point—applying pressure to the active TrP until the point was suppressed (turned off)—resulting in cessation of pain [6,7].

**Figure 4:**
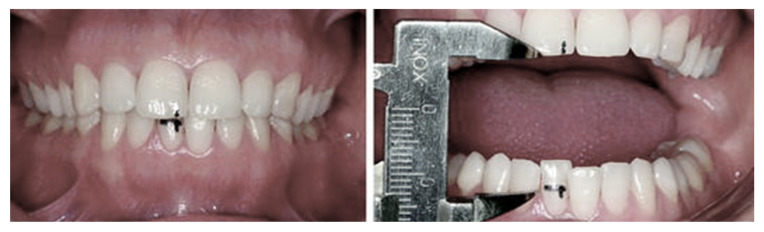
Maximal mandible abduction measurement.

In order to assess the stress-related increase in neuromuscular tension, maximal mandibular abduction measurements were made [9] with the use of an electronic caliper (mm), which was placed between the central incisors of the maxilla and mandible (Figure 4).

All measurements were conducted 3 times, and the mean value of each measurement was noted to exclude measurement error. Each of the tested subjects had maximal abduction measurements taken twice: before the mission and after the mission, and abduction range change was noted in the TMJ diagnosis aspect of the integrated dental examination card SZOPPDP©.

The mean value was calculated for all tested subjects from each of the mandible abduction measurements, and the standard deviation was noted. The difference in mean results between the 1st and 2nd measurement was compared and analyzed. The normal distribution was calculated with the use of a Shapiro–Wilk normality test. To conduct statistical analysis, a paired *t*-test (for paired data) and Welch’s Two-Sample *t*-test (for unpaired data) were used with Statistica (TIBECO) software. Statistical significance was calculated for both tested groups (*p*-value < 0.05); the abduction range change was compared in both groups.

The integrated dental examination card SZOPPDP© consists of an introductory part (I) containing personal and general medical data and 4 detailed parts (IIa–d), containing: oral cavity diagnosis, a questionnaire regarding dietary habits, psychological, and hygienic aspects. The dental part (IIa 1–4) includes a conservative-hygiene assessment, assessment of the masticatory system (TMJ-muscles-posture), and orthodontic and periodontological diagnoses. Part III contains tables for risk assessment taking into account pathogenic and preventive factors (IIIa) and personalized recommendations (IIIb) based on data collected in the SZOPPDP© diagnoses and surveys (parts I and II).

Patients, based on the data contained in SZOPPDP©, can be classified into appropriate high-risk groups for caries, gingivitis, orthodontic defects, temporomandibular joint dysfunction, or odontophobia. Based on the analysis of risk factors present, we identified a group of patients with higher TMD risk; in those cases, specialist consultation was recommended.

Classification of increased TMD risk was determined after the completion of the study based on data collected from the patient’s medical history and extra- and intra-oral examination. The selected criteria indicated pre-existing disturbances in TMJ function [10,11] as follows: presence of increased neuromuscular tension of masticatory muscles, clicking or jumping of TMJ, pain within masticatory or TMJ associated muscles, history of TMJ trauma, and/or occupational hazard (lack of movement, stress, forced working position).

## 3. Results

**Figure 5:**
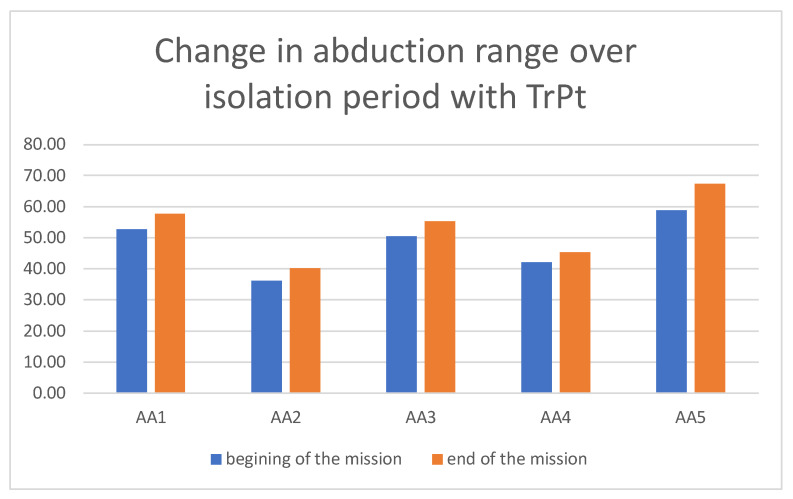
Change in maximal mandibular abduction range over isolation period in AA group with TrPt (test group).

An increase in mandible abduction range was observed in each subject of the test group as a result of decreased neuromuscular tension by TrPt (Figure 5).

**Figure 6:**
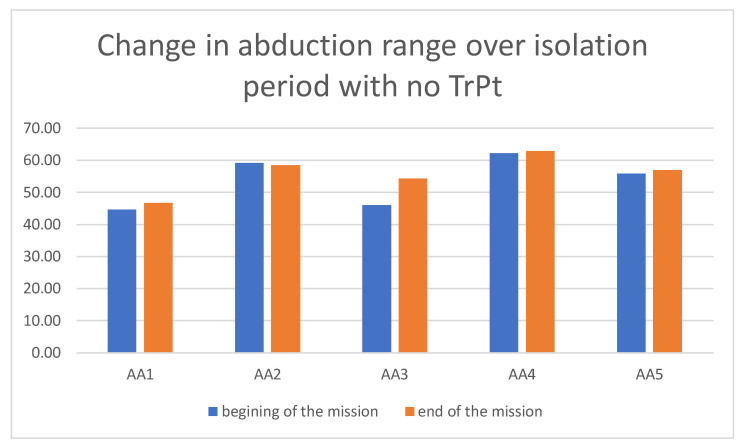
Change in maximal mandibular abduction range over isolation period in AA group with no TrPt (control group).

This pattern was not present in the group of AA who did not undergo TrPt during the mission (Figure 6).

**Figure 7:**
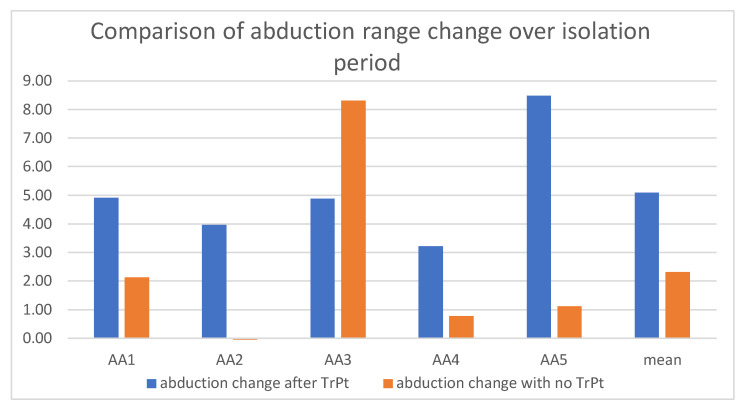
Comparison of maximal mandibular abduction range change over isolation period in both tested groups of AA.

In comparison of the abduction range for both groups, the test group had an overall greater increase (Figure 7).

The average maximal mandibular abduction range change (mm) in the group of AA who underwent TrPt during the mission was 5.09 mm (Table 2), in contrast to the control group, in which the mean abduction range change was 2.32 mm (Table 3).

Statistical analysis showed significance in differences in mandibular abduction range at the beginning and end of the mission in AA who underwent TrPt (*p* < 0.05) (Table 4); however, differences in mandibular abduction range over the isolation period between both groups of AA did not show significance.

Based on guidelines for temporomandibular disorders in children and adolescents of the American Academy of Family Physicians and American Academy of Pediatric Dentistry, limiting the extent of mandibular abduction is one of the risk factors for TMD, in addition to head and neck pain, mandibular dysfunction, history of orofacial trauma, or present illness with an account of current TMD symptoms. All of these factors were included in the SZOPPDP© card, and on its basis, AA were classified into appropriate TMD risk groups (Table 5). With the help of SZOPPDP©, it was possible to lay out and distribute individual recommendations, which included prevention remarks and/or treatment of temporomandibular dysfunction suggestions.

### Summary of the Results

1. TrPt enhanced the scope of mandibular abduction in all AA of the test group.
2. In the group of AA not receiving TrPt, no increased mandible abduction range was observed.
3. The observed change in the mandibular abduction range in the study group was greater than in the control group.
4. Based on risk assessment of TMD and diagnostic guidelines for temporomandibular disorders in children and adolescents of the American Academy of Family Physicians and American Academy of Pediatric Dentistry included in the SZOPPDP© card, AA with a high, medium, or low risk of TMD were diagnosed, and prophylactic and therapeutic recommendations were given accordingly.

## 4. Discussion

It was observed that permanent stress may increase neuromuscular tension. Lundberg observed increased neuromuscular tension of the trapezius muscle among patients subjected to long-term stress [12]. Our research has shown that the possible stress of 14 days of isolation did not affect the neuromuscular tension level. Measurements were conducted on a group of masseter muscles, and no increase in neuromuscular tension was observed. It cannot be ruled out that the conditions prevailing in LunAres (good atmosphere in the group, good organization of work, clearly defined common goals, precisely ordered daily rhythm) minimized the stress, as a result of which, no increase in the neuromuscular tension in the region of the masseter muscles was observed.

Research also conducted in the area of the neck and shoulder girdle suggests [13] that the increase in neuromuscular tension in this group of muscles is related to the overload caused by a forced working position [14,15,16,17]. In the case of the AA, efforts were made to avoid excessive strain on the muscles resulting from forced work positions by changing activities frequently according to a detailed daily schedule.

The group of AA subjected to the TrPt showed an increase in the range of maximal mandibular abduction caused by a decrease in neuromuscular tension [11]. Thus, the therapy should be recommended to people exposed to factors causing neuromuscular tension (stress, bruxism, hazardous occupation position, reduced physical activity, parafunctions, anxiety, depression) [10,17,18,19]. It seems that isolation as a single factor does not necessarily increase neuromuscular tension [20]. Our study should be performed on a larger group of AA.

Diagnosing patients with the use of the SZOPPDP**^®^** card allowed for identifying TMD high-risk patients and also recommending them for an appropriate specialist consult so they can seek further treatment.

## 5. Conclusions

1. Manual TrPt effectively decreases neuromuscular tension of the masseter muscle, which results in an increased mandibular abduction range of the AA.
2. Observations concluded in LunAres Research Station regarding stress-related neuromuscular tension can help identify effective therapeutic methods in circumstances of social isolation.
3. The research should be continued and confirmed on a greater number of cases, as well as on a different group of patients subjected to stressful situations affecting neuromuscular tension.
4. The integrated dental examination card SZOPPDP© was recognized as a useful tool for conducting a comprehensive dental diagnosis and qualifying stomatognathic high-risk groups to create personalized recommendations for each of the tested subjects.

## Figures and Tables

**Table 1:** Detailed information about the AA participating in the study.

Variables	Test Group	Control Group
Number of AA	5	5
Age (range)	24–32	23–34
Gender (F/M)	2F/3M	2F/3M
Education (elementary/secondary/higher)	Higher (all participants)	Higher (all participants)
Experience of participating in similar missions (number of AA/5 AA)	2/5	2/5

**Table 2:** Results of measurements of maximal mandibular abduction (mm) of test group—AA who underwent TrPt during the mission.

AA—Test Group (AAT)	AA1 (mm)	AA2 (mm)	AA3 (mm)	AA4 (mm)	AA5 (mm)	Mean (mm)	Standard Deviation
Beginning of the mission	52.77	36.16	50.41	42.09	58.88	48.06	8.97
End of the mission	57.68	40.12	55.29	45.31	67.36	53.15	10.70
Abduction change after TrPt	**4.91**	**3.96**	**4.88**	**3.22**	**8.48**	**5.09**	**5.13**

**Table 3:** Results of measurements of maximal mandibular abduction (mm) of control group—AA who did not undergo TrPt during the mission.

AA—Control Group (AAC)	AA1 (mm)	AA2 (mm)	AA3 (mm)	AA4 (mm)	AA5 (mm)	Mean (mm)	Standard Deviation
Beginning of the mission	44.66	59.21	46.08	62.22	55.88	53.61	7.87
End of the mission	46.79	58.45	54.39	63.00	57.00	55.93	5.99
Abduction change without TrPt	**2.13**	**−0.76**	**8.31**	**0.78**	**1.12**	**2.32**	**2.35**

**Table 4:** Statistical analysis with the use of paired *t*-test (for paired data) and Welch’s Two-Sample *t*-test (for unpaired data).

Statistical Analysis		Normal Distribution	Paired *t*-Test	Welch Two Sample *t*-Test
		*p*-Value	*p*-Value	*p*-Value
AAT	(X1) Beginning	0.8783	(x1 vs. y1) **0.004894**	(x1 vs. x2) **0.3294**
	(Y1) End	0.8792		
AAC	(X2) Beginning	0.3571	(x2 vs. y2) **0.2139**	(y1 vs. y2) **0.6303**
	(Y2) End	0.8318		

**Table 5:** Summary of risk assessment of TMD based on SZOPPDP© card and diagnostic guidelines for temporomandibular disorders in children and adolescents of the American Academy of Family Physicians and American Academy of Pediatric Dentistry.

TMD Diagnosed	Number of Participants
High risk	6
Medium risk	2
Low risk	2

## Data Availability

Not applicable.
